# MPEXS‐DNA, a new GPU‐based Monte Carlo simulator for track structures and radiation chemistry at subcellular scale

**DOI:** 10.1002/mp.13370

**Published:** 2019-01-22

**Authors:** Shogo Okada, Koichi Murakami, Sebastien Incerti, Katsuya Amako, Takashi Sasaki

**Affiliations:** ^1^ KEK 1‐1, Oho Tsukuba Ibaraki 305‐0801 Japan; ^2^ University of Bordeaux CENBG UMR 5797 Gradignan F‐33170 France; ^3^ CNRS IN2P3 CENBG UMR 5797 Gradignan F‐33170 France

**Keywords:** CUDA, Geant4‐DNA, GPGPU, microdosimetry, Monte Carlo simulation

## Abstract

**Purpose:**

Track structure simulation codes can accurately reproduce the stochastic nature of particle–matter interactions in order to evaluate quantitatively radiation damage in biological cells such as DNA strand breaks and base damage. Such simulations handle large numbers of secondary charged particles and molecular species created in the irradiated medium. Every particle and molecular species are tracked step‐by‐step using a Monte Carlo method to calculate energy loss patterns and spatial distributions of molecular species inside a cell nucleus with high spatial accuracy. The Geant4‐DNA extension of the Geant4 general‐purpose Monte Carlo simulation toolkit allows for such track structure simulations and can be run on CPUs. However, long execution times have been observed for the simulation of DNA damage in cells. We present in this work an improvement of the computing performance of such simulations using ultraparallel processing on a graphical processing unit (GPU).

**Methods:**

A new Monte Carlo simulator named MPEXS‐DNA, allowing high computing performance by using a GPU, has been developed for track structure and radiolysis simulations at the subcellular scale. MPEXS‐DNA physics and chemical processes are based on Geant4‐DNA processes available in Geant4 version 10.02 p03. We have reimplemented the Geant4‐DNA process codes of the physics stage (electromagnetic processes of charged particles) and the chemical stage (diffusion and chemical reactions for molecular species) for microdosimetry simulation by using the CUDA language. MPEXS‐DNA can calculate a distribution of energy loss in the irradiated medium caused by charged particles and also simulate production, diffusion, and chemical interactions of molecular species from water radiolysis to quantitatively assess initial damage to DNA. The validation of MPEXS‐DNA physics and chemical simulations was performed by comparing various types of distributions, namely the radial dose distributions for the physics stage, and the G‐value profiles for each chemical product and their linear energy transfer dependency for the chemical stage, to existing experimental data and simulation results obtained by other simulation codes, including PARTRAC.

**Results:**

For physics validation, radial dose distributions calculated by MPEXS‐DNA are consistent with experimental data and numerical simulations. For chemistry validation, MPEXS‐DNA can also reproduce G‐value profiles for each molecular species with the same tendency as existing experimental data. MPEXS‐DNA also agrees with simulations by PARTRAC reasonably well. However, we have confirmed that there are slight discrepancies in G‐value profiles calculated by MPEXS‐DNA for molecular species such as H_2_ and H_2_O_2_ when compared to experimental data and PARTRAC simulations. The differences in G‐value profiles between MPEXS‐DNA and PARTRAC are caused by the different chemical reactions considered. MPEXS‐DNA can drastically boost the computing performance of track structure and radiolysis simulations. By using NVIDIA's GPU devices adopting the Volta architecture, MPEXS‐DNA has achieved speedup factors up to 2900 against Geant4‐DNA simulations with a single CPU core.

**Conclusion:**

The MPEXS‐DNA Monte Carlo simulation achieves similar accuracy to Monte Carlo simulations performed using other codes such as Geant4‐DNA and PARTRAC, and its predictions are consistent with experimental data. Notably, MPEXS‐DNA allows calculations that are, at maximum, 2900 times faster than conventional simulations using a CPU.

## 1 Introduction

Modeling the biophysical processes associated with radiation‐induced cellular damage, eventually leading to cell death, is a complex challenge which requires a detailed description of the physical, chemical, and biological interactions of ionizing radiation with organic media.^1^ In particular, for more than 20 yr, track structure codes have been developed in order to accurately simulate physical interactions at the DNA and cellular scale, underlining the necessity to carefully simulate energy deposition for the prediction of critical biological lesions. Among the variety of track structure codes that have been developed so far, the Geant4‐DNA code, which is fully included in the Geant4 general‐purpose particle–matter Monte Carlo simulation toolkit,^2^, ^3^, ^4^ was the first to propose in open access a full set of features allowing the simulation of radiation physical interactions in liquid water as well as water radiolysis, in combination with geometrical models of biological targets for the simulation of early DNA damage.^5^, ^6^, ^7^ We recently demonstrated the possibility of simulating early damage in simplified models of bacteria^8^ and human cells.^9^, ^10^ However, such simulations require very significant computing times even on computer clusters, especially for the simulation of chemical reactions between molecular species created from water radiolysis in the irradiated geometrical model such as the full DNA content of a genome. As an example, the simulation of the induction of biological damage in a single cell irradiated with a thousand 1 MeV protons using Geant4‐DNA may take several days on a CPU cluster.^9^

Benefiting from recent progress in computing performance, the group of Sasaki et al. at KEK in Japan has proposed to migrate Geant4‐DNA to graphics processing units (GPUs). They addressed the porting of Geant4‐DNA modeling of physical interactions in liquid water to GPUs, reaching almost two orders of magnitude in performance gain.^11^, ^12^ These developments have now been extended in order to accelerate very significantly the simulation of water radiolysis, a key requirement for the fast simulation of DNA damage induced indirectly by molecular species.

This paper describes the development of the MPEXS‐DNA simulation code, based on Geant4‐DNA and fully deployed on GPU architecture. In Section Methods, we first recall the principle of modeling ionizing radiation in physical and chemical processes in liquid water, the main component of biological medium. We then describe how such processes have been implemented in MPEXS‐DNA and verify their implementation by comparison to Geant4‐DNA simulations of absorbed radial dose distributions around ion tracks and radiochemical yields as a function of time. In Section Results, comparisons to experimental data on radial doses and chemical yields as a function of time and linear energy transfer (LET) are presented as well as the results of validation of the code. Finally, gains in terms of computing performance are presented. MPEXS‐DNA allows a speedup of three orders of magnitude compared to a single CPU approach.

## 2 Materials and methods

### 2.A. Overview of track structures and water radiolysis simulations

Initial damage to DNA caused by radiation is classified as either direct or indirect. Electromagnetic interactions (e.g., ionization and excitation) occur along the tracks of charged particles when they pass through a cell. Owing to the excess energy, the DNA molecules become unstable, and their molecular bonds are thus severed. Such damage is classified as direct since it results directly from the radiation. These electromagnetic interactions also induce radiolysis of liquid water molecules, the main component of the cellular medium. During water radiolysis, molecular species such as oxidative radicals and ions are created. The damaging effects of these molecular species on DNA molecules are classified as indirect. The Monte Carlo simulation of DNA damage from ionizing radiation is classically divided into four stages^13^: (1) the physics stage, (2) the physicochemical stage, (3) the chemical stage, and (4) the biological stage. These stages allow a quantitative assessment of the induced DNA damage through the mechanistic simulation of physical interactions and liquid water radiolysis. An overview of the four stages is given below.

#### 2.A.1. The physical stage

During this stage, the electromagnetic interactions between charged particles and liquid water molecules are simulated, and the distribution of energy loss in the cellular medium, approximated as liquid water, is calculated. Quantitative measurement of the direct damage to DNA requires the accurate simulation of local energy loss distributions. Thus, all physical responses are simulated and are treated as “discrete” processes, allowing step‐by‐step description of track structures in small volumes (e.g., nanometer) and down to very low energy (e.g., a few eV). Physical processes such as ionization, excitation, and dissociative electron attachment produce ionized (H_2_O^−/+^) and excited water molecules (H_2_O*) as well as hydrated electrons ($e_{\mathrm{aq}}^{-}$).

#### 2.A.2. The physicochemical stage

In this stage, the dissociation of H_2_O* molecules and H_2_O^−/+^ ions by electronic ionization and excitation of water molecules during the physics stage is simulated, resulting in the production of other molecular species such as radicals, ions, and molecules.

#### 2.A.3. The chemical stage

For molecular species like ions and free radicals which are generated during the physicochemical stage, diffusion and mutual chemical reactions (leading to the production of other molecular species) are simulated. The physicochemical stage is considered to occur within 1 ps after the irradiation of the liquid medium. The chemical stage takes place from 1 ps up to 1 μs. Time profiles of the distributions of molecular species in the target can be simulated.

#### 2.A.4. The biological stage

A quantitative estimate of initial DNA damage can be made using the distribution of the energy loss and molecular species produced in the irradiated medium.

### 2.B. MPEXS, the Massive parallel electrons and x ray simulator

**Figure 1:**
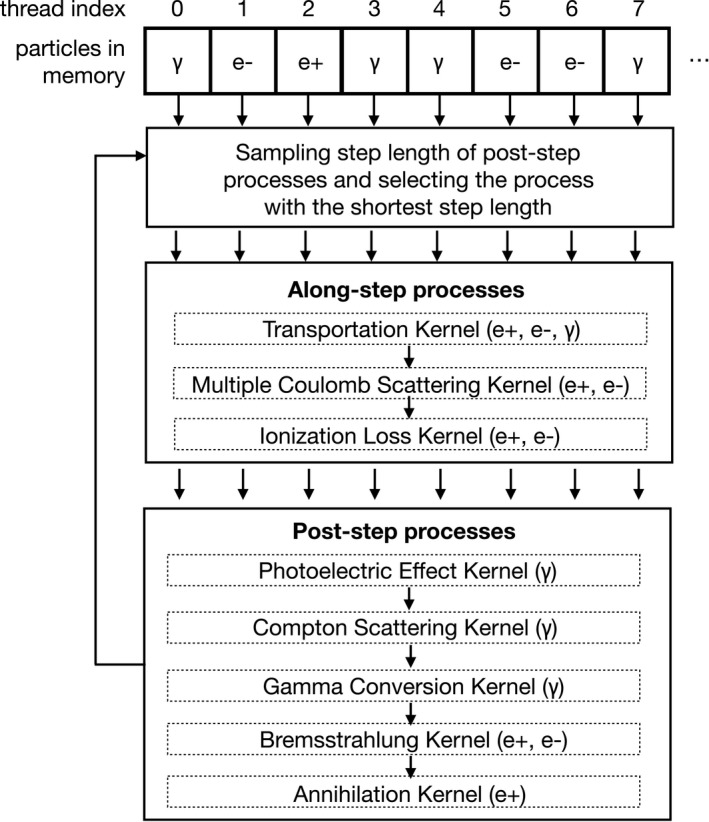
The diagram of parallel particle tracking in MPEXS simulation.

MPEXS, which is the core software of MPEXS‐DNA, is a radiation simulator that utilizes a GPU. Standard electromagnetic interactions of Geant4 10.02 p03^2^, ^3^, ^4^ for electrons, positrons, and photons have been reimplemented in the CUDA language.^14^, ^15^ MPEXS was developed for practical use as a high‐speed dose calculation engine for cancer radiotherapy. MPEXS can handle computed tomography images that are reconstructed with voxelized geometry in the simulation, and assign water‐equivalent material to each voxel to perform a dose calculation. In an MPEXS simulation, each GPU thread tracks a charged particle, and the amount of energy loss that occurs in each material is accumulated in voxel units. GPU devices equip several thousands of computation cores. MPEXS creates several millions of GPU threads in these cores, which allows ultraparallel tracking of particles using the Monte Carlo method. Figure 1 shows a diagram of parallel particle tracking in the MPEXS framework. MPEXS launches CUDA kernels of physics processes at every iteration, and GPU threads track particles. First, the step length for each post‐step process is sampled by using cross‐sectional data, and the next interaction point is selected with the shortest step length. Transportation, multiple Coulomb scattering, and ionization loss are executed as along step processes. Then, kernel functions of post‐step processes are serially applied and threads simulate the selected physical processes. Because each thread processes a different kernel of physics process selected at every iteration, thread divergence is observed. However, thread efficiency of about 50% has been measured. MPEXS performs dosimetry simulations of the standard electromagnetic interactions in water with the same accuracy as that of Geant4, and it achieves speeds that are, at maximum, 700 times faster than Geant4 simulations using a single CPU core. Currently, MPEXS can simulate only electromagnetic physics for electrons and photons. We will extend the physics capability to hadrons and neutrons and apply it to the simulation of particle therapy in the future.

### 2.C. Track structures and radiolysis simulations by MPEXS‐DNA

MPEXS‐DNA has been developed as an extension of MPEXS for the simulation of ionizing radiation effects at the DNA scale. It currently simulates the three stages described in Section 2.A: (1) the physical stage, (2) the physicochemical stage, and (3) the chemical stage. The biological stage is not yet implemented in MPEXS‐DNA. The processing of the three stages by MPEXS‐DNA essentially follows the approach employed in the Geant4‐DNA extension package designed to perform track structures and radiolysis simulations available in Geant4 10.02 p03. Details of the simulation of each phase are given in the following paragraphs.

#### 2.C.1 The physical stage

For a quantitative estimation of direct DNA damage, the distribution of energy loss caused by charged particles is calculated during the physical stage. To assess where the charged particles deposit energy and cause damage in a DNA, it is necessary to simulate the track structures of each charged particle and associated secondary particles in a discrete (step‐by‐step) mode, followed by the calculation of the local energy loss distribution. The faster “condensed history” alternative approach, which groups several physical interactions (such as ionization and multiple Coulomb scattering), is not suitable for track structure simulations.^16^ In brief, the “condensed history” approach is largely adopted for general‐purpose Monte Carlo simulations of particle–matter interactions. It simplifies the simulation of physical interactions that occur along steps as the particle propagates through the medium, by calculating the amount of energy lost by the charged particle using stopping power information for the traversed material and step length. As a result, the spatial accuracy is degraded. Instead, all physical interactions are treated as discrete processes in MPEXS‐DNA simulation. Treating a physical interaction, such as ionization and excitation, as a discrete process allows the spatial accuracy to be preserved. MPEXS‐DNA adopts the same physical interactions that are available in the Geant4‐DNA extension of Geant4 (version 10.02 p03).^11^, ^12^ Charged particles handled in MPEXS‐DNA consist of electrons, protons, neutral hydrogen atoms, helium atoms with three charged states (He^0^, He^+^, He^++^), and ions (Li, Be, B, C, N, O, Si, Fe). Physical interactions for each particle, corresponding class names of Geant4‐DNA processes, and applicable energy ranges are shown in Tables 1, 2, 3, 4, 5, 6. MPEXS takes into consideration the following five physical interactions for electrons: elastic scattering, electronic excitation, ionization, vibrational excitation, and dissociative attachment. Regarding protons, neutral hydrogen atoms, and helium atoms with three charged states, the following four interactions are considered: elastic scattering, electronic excitation, ionization, and charge increase/decrease. Only ionization is considered for ions. During the ionization process, the simulation of atomic deexcitation is also taken into account (leading to the production of Auger electrons and characteristic x rays). The following four interactions are considered for x rays: the photoelectric effect, Compton scattering, electron–positron pair creation, and Rayleigh scattering. Each particle is transported down to a “tracking cut,” below which the tracking is stopped and the particle deposits its kinetic energy locally in the liquid water medium (see Table 7). Tracking is also stopped when the particle leaves the geometry. GPU devices allow MPEXS‐DNA to perform simultaneous parallel tracking of several millions of particles, which dramatically improves the computing performance of such simulations. MPEXS‐DNA currently handles only liquid water material, the main component of biological media. Cross sections of all physical interactions are applicable to liquid water and can be scaled to any density different than 1 g/cm^3^ if needed.

**Table 1:** Electromagnetic physics processes for electrons

Physical process	Reference class in Geant4‐DNA	Energy range
Elastic scattering	G4DNAUeharaScreenedRutherfordElasticModel	9 eV–10 keV
Electronic excitation	G4DNAEmfietzoglouExcitationModel	8 eV–10 keV
	G4DNABornExcitationModel	9 eV–1 MeV
Ionization	G4DNAEmfietzoglouIonisationModel	10 eV–10 keV
	G4DNABornIonisationModel	11 eV–1 MeV
Vibrational excitation	G4DNASancheExcitationModel	2 eV–100 eV
Dissociative attachment	G4DNAMeltonAttachmentModel	4 eV–13 eV

**Table 2:** Electromagnetic physics processes for protons

Physical process	Reference class in Geant4‐DNA	Energy range
Electronic excitation	G4DNAMillerGreenExcitationModel	10 eV–500 keV
	G4DNABornExcitationModel	500 keV–100 MeV
Ionization	G4DNARuddIonisationExtendedModel	0 eV–500 keV
	G4DNABornIonisationModel	500 keV–100 MeV
Charge increase	G4DNADingfelderChargeIncreaseModel	100 eV–100 MeV

**Table 3:** Electromagnetic physics processes for hydrogen atoms

Physical process	Reference class in Geant4‐DNA	Energy range
Electronic excitation	G4DNAMillerGreenExcitationModel	10 eV–500 keV
Ionization	G4DNARuddIonisationExtendedModel	0 eV–100 MeV
Charge increase	G4DNADingfelderChargeIncreaseModel	100 eV–100 MeV

**Table 4:** Electromagnetic physics processes for helium atoms with three charged states (He^++^, He^+^, He^0^)

Physical process	Reference class in Geant4‐DNA	Energy range
Electronic excitation	G4DNAMillerGreenExcitationModel	1 keV–400 MeV
Ionization	G4DNARuddIonisationExtendedModel	0 keV–400 MeV
Charge increase^a^	G4DNADingfelderChargeIncreaseModel	1 keV–400 MeV
Charge decrease^b^	G4DNADingfelderChargeDecreaseModel	1 keV–400 MeV

a Charge increase applies to He^0^ and He^+^ only.

b Charge decrease applies to He^++^ and He^+^ only.

**Table 5:** Electromagnetic physics processes for Li, Be, B, C, N, O, Si, and Fe ions

Physical process	Reference class in Geant4‐DNA	Energy range
Ionization	G4DNARuddIonisationExtendedModel	0.5 MeV/u–1.0 × 10^6^ MeV/u

**Table 6:** Physics processes for fluorescence x rays generated from deexcitation processes

Physical process	Reference class in Geant4	Energy range
Compton scattering	G4LivermoreComptonModel	100 eV–1 GeV
Gamma conversion	G4LivermoreGammaConversionModel	100 eV–1 GeV
Photoelectric effect	G4LivermorePhotoelectricModel	100 eV–1 GeV
Rayleigh scattering	G4LivermoreRayleighModel	100 eV–1 GeV

**Table 7:** Energy threshold (“tracking cuts”) below which tracking is stopped during the physical stage

Particle type	Energy threshold
Electrons	7.4 eV
Protons and neutral hydrogen atoms	100 eV
Helium atoms (He^0^, He^+^, He^++^)	1 keV
Ions (Li, Be, B, C, N, O, Si, and Fe)	0.5 MeV/u

#### 2.C.2 The physicochemical stage

H_2_O* and H_2_O^−/+^ are produced by excitation, ionization, and dissociative electron attachment. These molecules get converted to other molecular species such as radicals and ions as a result of this physicochemical stage.

H_2_O* decays by dissociation into other molecular species at 1 fs after irradiation. The decay channel depends on the excited state of the water molecule. In addition, the excess energy of H_2_O* is externally released and a shift to the ground state occurs (relaxation). H_2_O^+^ generated by the ionization process exchanges protons with other nearby water molecules within 10 fs after orbital electrons are stripped away. Then, the $H_{2}O^{+}+H_{2}O\to H_{3}O^{+}+\cdot \text{OH}$ reaction results in the production of H_3_O^+^ and ·OH. Table 8 shows the branching ratio of each dissociation and relaxation channel for excited molecules, ionized molecules, and the molecular species produced. The same parameters as Geant4‐DNA^13^, ^17^ are implemented in MPEXS‐DNA. The symbol “ΔE” in Table 8 indicates that excess energy is externally released when relaxation causes a shift to the ground state. These molecular species produced via dissociation repeats thermal motion releasing their own kinetic energy. MPEXS‐DNA follows Geant4‐DNA for the calculation of the migration distance by thermalization of molecular species.

**Table 8:** Dissociation channels of excited and ionized water molecules during the physicochemical stage of MPEXS‐DNA

Electronic state	Process	Dissociation channel	Fraction (%)
Ionization state	Dissociative decay	$H_{3}O^{+}+\cdot \text{OH}$	100
Excitation state: A1B1	Dissociative decay	·OH+H·	65
	Relaxation	H_2_O + ΔE	35
Excitation state: B1A1	Auto ionization	$H_{3}O^{+}+\cdot \text{OH}+e_{\mathrm{aq}}^{-}$	55
	Dissociative decay	$\cdot \text{OH}+\cdot \text{OH}+H_{2}$	15
	Relaxation	H_2_O + ΔE	30
Excitation state: Rydberg, diffusion bands	Auto ionization	$H_{3}O^{+}+\cdot \text{OH}+e_{\mathrm{aq}}^{-}$	50
	Relaxation	H_2_O + ΔE	50
Dissociative attachment	Dissociative decay	$\cdot \text{OH}+{\text{OH}}^{-}+H_{2}$	100

The tracking of electrons is stopped when the kinetic energy of electrons falls to 8.22 eV (the lowest excitation energy of liquid water molecule). Then, such electrons start to thermalize and become surrounded by water molecules forming hydrated electrons ($e_{\mathrm{aq}}^{-}$).

#### 2.C.3 The chemical stage

**Figure 2:**
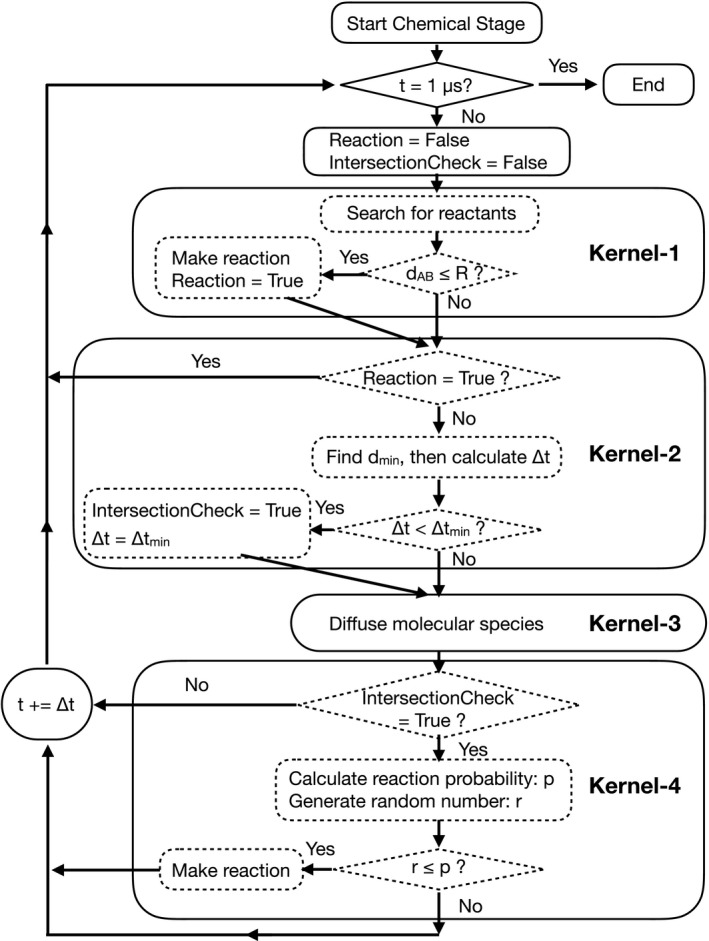
The process flow diagram of the MPEXS‐DNA chemical stage.

During the chemical stage, diffusion and chemical reactions of molecular species produced in the previous physical and physicochemical stages are considered. Molecular species are produced within 1 ps after irradiation in the physicochemical stage. Then, these molecular species repeat diffusions and chemical reactions in the medium. Early DNA damage by molecular species, well known as indirect effects, is considered to occur within 1 μs. We set the start and the end time of the chemical stage to 1 ps and 1 μs, respectively, as Geant4‐DNA does. Figure 2 shows the process flow diagram for the MPEXS‐DNA chemical stage. The simulation process consists of four steps. In each step, the corresponding GPU kernel function is executed on a GPU device. MPEXS‐DNA repeats these four steps until the end time (t = 1 μs). The formulae used to compute reaction radius and diffusion time (discussed below) are the same as those used by Geant4‐DNA.^13^, ^17^

##### Step 1: Searching for possible reactant pairs and making chemical reactions in Kernel‐1

The process of the MPEXS‐DNA chemical stage finds all possible reactant pairs in the simulation space. For each pair (molecular species A and B), the intermolecular distance (*d*_*AB*_) and reaction radius (*R*) of the corresponding chemical reaction are calculated. The reaction radius is given by the Smoluchowski model as follows^17^:

$$R=\frac{k}{4\pi N_{A}D_{\mathrm{sum}}}.$$

Here, *N*_*A*_ is Avogadro's constant, *k* is the reaction rate constant, and *D*_*sum*_ is the sum of the diffusion coefficients for A and B. The chemical reactions and corresponding reaction rate constants for the chemical stage are shown in Table 9, and diffusion coefficients for all molecular species are shown in Table 10. In the simulation process, a chemical reaction between A and B occurs when the condition *d*_*AB*_ *< R* is satisfied, and then replaces A and B with the molecular products C and D. This process is applied for all molecular species pairs at each time step of the simulation. The processing time required increases as O (*N* ^2^/2), where N is the total number of molecular species in the irradiated medium. Geant4‐DNA and gMicroMC^18^ divide the simulation geometry into grids in order to reduce the number of possible reactant pairs of molecular species. This reduces the computational time for searching pairs of molecular species. Nevertheless, the grid approach requires the update of a lookup table, which is a list of molecular species inside each grid, at every time step. This part is potentially quite time‐consuming. MPEXS‐DNA adopts a direct search for all possible pairs of molecular species in the simulation region. This direct approach does not require lookup tables and is suitable for parallel processing on GPU devices. Each GPU thread performs the search for molecule pairs. Sufficient GPU threads are set for the number of molecular pairs. Thus, the calculation time for the search does not depend on the number of molecular pairs.

**Table 9:** List of chemical reactions available in MPEXS‐DNA and corresponding reaction rate constants

Chemical reactions	Reaction rate constants
$2e_{\mathrm{aq}}^{-}+2H_{2}O\to H_{2}+2{\text{OH}}^{-}$	5.00 × 10^9^ dm^3^/(mol·s)
$e_{\mathrm{aq}}^{-}+\cdot \text{OH}\to {\text{OH}}^{-}$	2.95 × 10^10^ dm^3^/(mol·s)
$e_{\mathrm{aq}}^{-}+H\cdot +H_{2}O\to {\text{OH}}^{-}+H_{2}$	2.65 × 10^10^ dm^3^/(mol·s)
$e_{\mathrm{aq}}^{-}+H_{3}O^{+}\to H\cdot +H_{2}O$	2.11 × 10^10^ dm^3^/(mol·s)
$e_{\mathrm{aq}}^{-}+H_{2}O_{2}\to {\text{OH}}^{-}+\cdot \text{OH}$	1.41 × 10^10^ dm^3^/(mol·s)
$\cdot \text{OH}+\cdot \text{OH}\to H_{2}O_{2}$	4.40 × 10^9^ dm^3^/(mol·s)
$\cdot \text{OH}+H\cdot \to H_{2}O$	1.44 × 10^10^ dm^3^/(mol·s)
$H\cdot +H\cdot \to H_{2}$	1.20 × 10^10^ dm^3^/(mol·s)
H_3_O^+^ + OH^−^ → 2H_2_O	1.43 × 10^11^ dm^3^/(mol·s)

**Table 10:** List of molecular species considered in MPEXS‐DNA and corresponding diffusion coefficients for liquid water

Molecular species	Diffusion coefficient
H_3_O^+^	9.0 × 10^−9^ m^2^/s
H·	7.0 × 10^−9^ m^2^/s
OH^−^	5.0 × 10^−9^ m^2^/s
$e_{\mathrm{eq}}^{-}$	4.9 × 10^−9^ m^2^/s
H_2_	5.0 × 10^−9^ m^2^/s
·OH	2.8 × 10^−9^ m^2^/s
H_2_O_2_	1.4 × 10^−9^ m^2^/s

##### Step2: Computing a diffusion time in Kernel‐2

Next, a search is made for the minimum *d*_*min*_ among the *d*_*AB*_ distances calculated in step (1). The time step Δ*t* is calculated using the formula shown below:

$$\Delta t=\frac{{\left(d_{\min}-R\right)}^{2}}{8\left(D_{A}-D_{B}+2\sqrt{D_{A}D_{B}}\right)}$$

where *D*_*A*_ and *D*_*B*_ are the diffusion coefficients corresponding to the molecular species, and *R* is the reaction radius of a chemical reaction that occurs in each pair. The Δ*t* value calculated using this formula guarantees that, for the processing of diffusion during the Δ*t* interval, none of the molecular species will be closer than the reaction radius.^17^ Just after the simulation process shifts from the physics stage to the chemical stage, molecular species are concentrated along the track of an incident charged particle. Intermolecular distances between the molecular species are small, and as a result, the time step Δ*t* is shortened and the processing time for the chemical stage becomes longer. To avoid increases in the simulation time, the minimum time step Δ*t*_*min*_ is set to 1 ps. In cases where the result of the time step calculation by using the above formula is Δ*t *< Δ*t*_*min*_, the time step for molecular species diffusion is set to Δ*t* = Δ*t*_*min*_ (=1 ps).

##### Step3: Diffusing molecular species in Kernel‐3

The time step Δ*t* calculated in step (2) is utilized to diffuse all molecular species in the simulation region. The direction of diffusion is randomly sampled. All molecular species in the simulation space are spread over GPU threads and diffused in parallel. For species that move beyond the simulation region during diffusion, the chemical stage simulation processing is stopped at that point. The possibility that they return to the simulation region is not considered. The mean free path for each molecular species during the time interval of 1 μs is estimated to be 50–130 nm from the diffusion coefficients in Table 10. These are much smaller than the size of the cell nucleus (typical size: 10–30 μm). Almost all molecular species stay in the simulation geometry, and those leaving the geometry can be ignored.

##### Step 4: Intersection checking in Kernel‐4

If Δ*t* < Δ*t*_*min*_ is observed and the diffusion time is set to Δ*t* = 1 ps, there is a possibility that diffusion paths of molecular species intersect on the way and some chemical reactions might happen during diffusion. The probability that a reaction involving molecular species A and B occurs during diffusion can be estimated by using the following formula^17^:

$$p=\exp\left(\frac{d_{i}\cdot d_{f}}{\left(D_{A}+D_{B}\right)\cdot \Delta t}\right).$$

Here, *d*_*i*_ and *d*_*f*_ are the intermolecular distances between the molecular species A and B before and after diffusion, respectively. In cases of Δ*t* < Δ*t*_*min*_ as a result of step (2), the probabilities of chemical reactions occurring during the diffusion process are estimated for all molecular species pairs in the simulation region after step (3). Then, whether reactions will occur is determined by random sampling based on the probabilities.

#### 2.C.4. Verification of MPEXS‐DNA by comparison to Geant4‐DNA simulations

##### Physics state simulation

**Figure 3:**
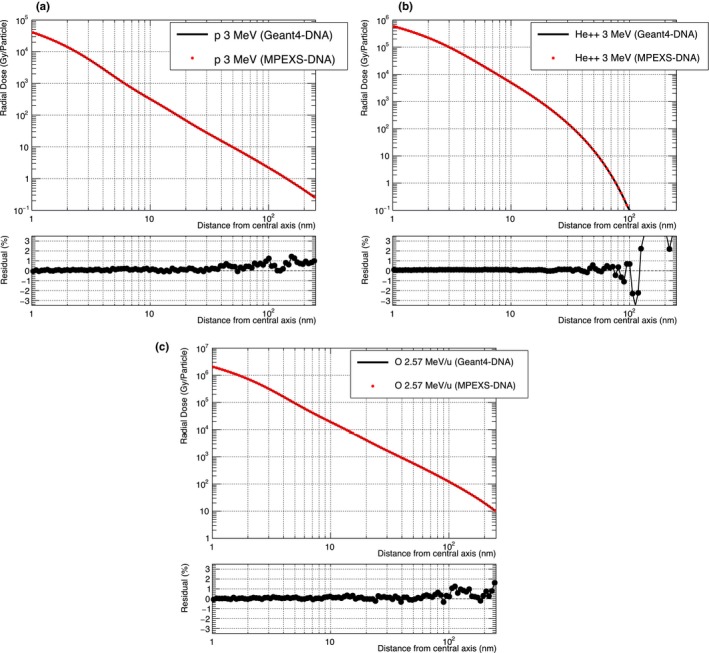
Comparison of radial dose between Geant4‐DNA 10.02 p03 (solid black line) and MPEXS‐DNA (filled red circle) for protons (a) and alpha particles (b) with kinetic energy 3 MeV, and 2.57 MeV/u oxygen ions (c). The bottom of each plot shows the residual of radial dose between Geant4‐DNA and MPEXS‐DNA.

As part of the verification of the MPEXS‐DNA physics process, we compared the simulation of radial dose distributions with Geant4‐DNA. Initial particles were set to 3 MeV protons and alpha particles and 2.57 MeV/u oxygen particles. Figure 3 shows the radial dose distributions for all cases. In this figure, the red circles indicate the results obtained with MPEXS‐DNA, and the solid black lines show the results for Geant4‐DNA. The doses on the vertical axis in Fig. 3 are normalized per incident particle. In all cases, MPEXS‐DNA is consistent with Geant4‐DNA within a 1% accuracy.

##### Chemical stage simulation

**Figure 4:**
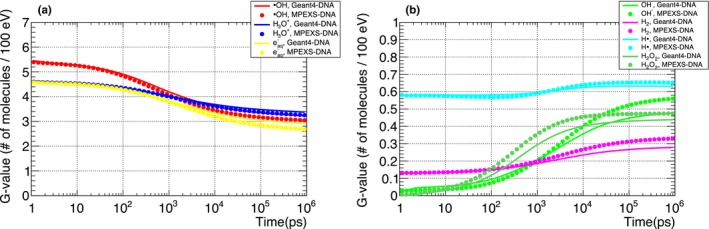
Comparison of G‐value time profiles for each molecular species induced by electrons with kinetic energy 750 keV. ·OH radicals, H_3_O^+^ ions, and hydrated electrons are shown in (a). OH^−^ ions, H· radicals, H_2_ and H_2_O_2_ molecules are shown in (b). Each color denotes a molecular species. Solid lines are Geant4‐DNA 10.02 p03 results and filled circles are MPEXS‐DNA values.

**Figure 5:**
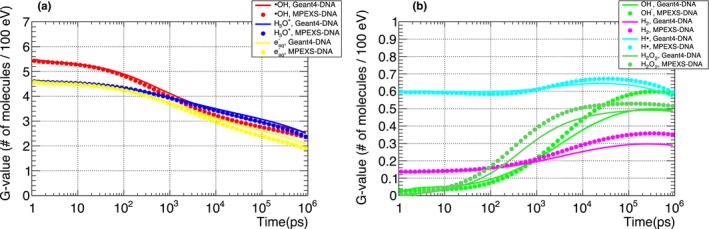
Comparison of G‐value time profiles for each molecular species induced by 20 MeV protons. ·OH radicals, H_3_O^+^ ions, and hydrated electrons are shown in (a). OH^−^ ions, H· radicals, H_2_, and H_2_O_2_ molecules are shown in (b). Each color indicates a molecular species. Solid line and filled circle plots are simulation results by Geant4‐DNA 10.02 p03 and MPEXS‐DNA, respectively.

G‐value time profiles were compared with Geant4‐DNA to verify the MPEXS‐DNA chemical stage simulation. G‐value is defined as the number of molecular species for an energy deposition of 100 eV into the medium. Figures 4 and 5 show the time profiles between 1 ps and 1 μs after irradiation for the molecular species produced when the target is irradiated with 750 keV electrons and 20 MeV protons. In Tables 11 and 12, we show the G‐values for all molecular species at 1 ps, 1 ns, and 1 μs after irradiation. At 1 ps after irradiation, that is, just after the start of the chemical stage, no difference is observed between the G‐values obtained by Geant4‐DNA and MPEXS‐DNA. Thus, the production of molecular species by MPEXS‐DNA through physical and physicochemical stages works correctly. After 1 ns, differences in the G‐value profiles are observed. This is particularly marked at t = 1 μs (simulation end point). G‐values of hydrated electrons, ·OH radicals, and H_3_O^+^ ions calculated by MPEXS‐DNA simulations are lower than Geant4‐DNA but higher for the other molecular species. As described in Section 2.C.3, the occurrence of a chemical reaction is obtained by comparisons of the distance between the molecular species to reaction radii. The time required for this process increases with the number of molecular species in the irradiated target. In Geant4‐DNA, the simulation region is divided into smaller areas to limit the range of search for molecular species pairs. The number of pairs searched is thus reduced and processing time is shortened. However, this method can miss some chemical reactions. In MPEXS‐DNA, chemical reactions are determined based on all combinations, which prevent missing any chemical reactions. MPEXS‐DNA tends to have a higher occurrence rate for chemical reactions than Geant4‐DNA. In fact, hydrated electrons, ·OH radicals, and H_3_O^+^ ions undergo chemical reactions transforming to OH^−^ ions and H_2_O_2_ molecules more than in Geant4‐DNA, and thus, differences in G‐values are observed in Figs. 4(b) and 5(b).

**Table 11:** Comparison of G‐values for each molecular species at 1 ps, 1 ns, and 1 μs after irradiation for 750 keV electrons (Geant4‐DNA 10.02 p03 vs MPEXS‐DNA)

	t = 1 ps		t = 1 ns		t = 1 μs	
	Geant4‐DNA	MPEXS‐DNA	Geant4‐DNA	MPEXS‐DNA	Geant4‐DNA	MPEXS‐DNA
·OH	5.45	5.45	4.16	4.02	3.28	3.04
$e_{\mathrm{aq}}^{-}$	4.62	4.61	3.80	3.76	2.90	2.67
H_3_O^+^	4.63	4.63	4.00	3.98	3.34	3.25
H·	0.58	0.58	0.60	0.59	0.63	0.65
H_2_	0.13	0.13	0.19	0.20	0.28	0.33
OH^−^	0.02	0.03	0.21	0.21	0.48	0.56
H_2_O_2_	0	0	0.30	0.36	0.44	0.47

**Table 12:** Comparison of G‐values for each molecular species at 1 ps, 1 ns, and 1 μs after irradiation for 20 MeV protons (Geant4‐DNA 10.02 p03 vs MPEXS‐DNA)

	t = 1 ps		t = 1 ns		t = 1 μs	
	Geant4‐DNA	MPEXS‐DNA	Geant4‐DNA	MPEXS‐DNA	Geant4‐DNA	MPEXS‐DNA
·OH	5.49	5.48	4.07	3.92	2.49	2.30
$e_{\mathrm{aq}}^{-}$	4.61	4.61	3.70	3.67	2.02	1.85
H_3_O^+^	4.64	4.64	3.93	3.91	2.42	2.29
H·	0.60	0.60	0.62	0.61	0.56	0.58
H_2_	0.14	0.14	0.21	0.21	0.29	0.35
OH^−^	0.02	0.02	0.23	0.22	0.49	0.58
H_2_O_2_	0	0	0.34	0.40	0.48	0.51

### 2.D. Validation of MPEXS‐DNA

MPEXS‐DNA simulations have been validated for the physical and chemical stages. The results of each stage have been compared with existing experimental data and calculations using other simulation codes. Both were consistent with MPEXS‐DNA simulations as a result. The simulations performed for validating MPEXS‐DNA are described below.

#### 2.D.1. MPEXS‐DNA physics validation

The physics stage of MPEXS‐DNA was validated through comparison with radial dose distributions. The scoring region was defined as multiple cylindrical shell‐shaped layers. The initial particle momenta were along the central axis of the cylinder, and the energy loss was accumulated into each layer to obtain the radial dose distribution. Various parameters for the dose scoring region are listed in Table 13. The scoring region was chosen to be an area 1–250 nm from the central axis, and this space was then divided into multiple layers using a logarithmic scale. The total number of layers was set to 100. The height of the cylinder in the scoring region was selected as 10 μm. The particles used for irradiation were protons, alpha particles, and oxygen ions. The kinetic energy was set to 3 MeV for protons and alpha particles and to 2.57 MeV/u for each nucleus of the oxygen ions. The radial dose distributions calculated from MPEXS‐DNA simulations were compared to existing experimental data^19^, ^20^ and to numerical calculations proposed by Chunxiang et al.^21^ and Waligórski et al.^22^

**Table 13:** Parameters of the scoring region for energy deposition

Inner radius	1 nm
Outer radius	250 nm
Height of cylinder scoring dose	10 μm
Number of shells in scoring region	100

#### 2.D.2. MPEXS‐DNA chemistry validation

The chemical stage allows the calculation of the time evolution of G‐values of molecular species generated from radiolysis of water molecules. The G‐value is defined as the number of molecular species as a result of energy deposition of 100 eV to a given substance:

$$\text{G‐value}=\frac{\text{Number of molecular species generated}}{\text{Energy deposition of 100 eV}}.$$

In this study, the time profiles of G‐values were estimated when the water targets were irradiated with 750 keV electrons and with 5 and 20 MeV protons, respectively. Then, MPEXS‐DNA simulation results were compared with experimental data and simulations performed by PARTRAC^23^ to validate chemical process simulations by MPEXS‐DNA. Track structure simulations have been an active area of research in the last decades. Many codes have been developed, especially for radiobiology applications, such as PARTRAC, KURBUC,^24^ NOREC^25^ codes, and others.^26^, ^27^ The modeling of the chemical stage in Geant4‐DNA followed the approach of PARTRAC thanks to the help Dr. Werner Friedland. Thus, we chose PARTRAC as the reference for validations of the MPEXS‐DNA chemistry stage. We conducted simulations using the conditions listed in Table 14. The G‐values were calculated by using total energy deposition by charged particles in the target and the number of molecular species at each time step.

**Table 14:** Simulation criteria for validation of MPEXS‐DNA chemical stage

Particle type	Electrons	Protons	Protons
Incident energy	750 keV	5 MeV	20 MeV
Target size	20 × 20 × 20 μm^3^	1 × 1 × 1 μm^3^	1 × 1 × 1 μm^3^

In addition, LET dependency of the G‐values was estimated for each molecular species. Here, a water target of 1 × 1 × 1 μm^3^ was irradiated with protons with kinetic energy between 500 keV and 100 MeV. In each case, the G‐value for each molecular species was calculated at 1 μs after irradiation (at the end point of the chemical stage simulation). LET was calculated from the mean value of energy deposition in the cube‐shaped liquid water target. Then, the results of MPEXS‐DNA simulations were compared with existing experimental data as well as PARTRAC and other simulations.

## 3 Results and discussion

### 3.A. Validation of the physics stage of MPEXS‐DNA

**Figure 6:**
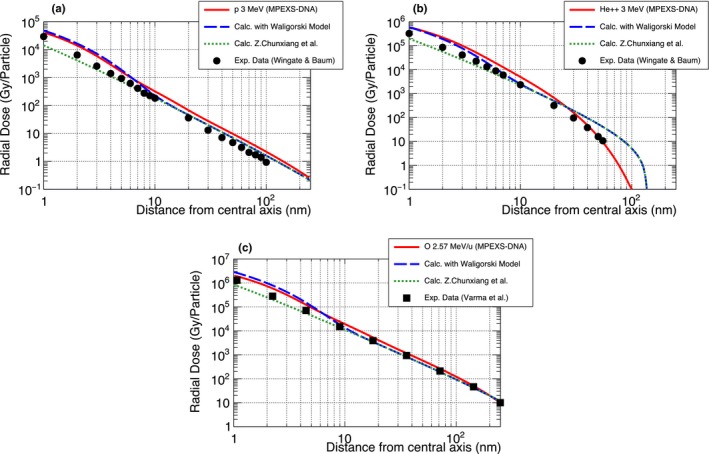
Comparison of radial dose between Monte Carlo simulation by MPEXS‐DNA (solid red line), numerical calculations by the Waligórski (dashed blue line)^22^ and Chunxiang et al. (dotted green line)^21^ models, and experiment data (filled circle^19^ and filled square^20^). Three types of initial particles are considered: 3 MeV protons (a) and alpha particles (b), 2.57 MeV/u oxygen ions (c).

Comparisons of the radial dose distributions for protons with 3 MeV kinetic energy are shown in Fig. 4(a). In this figure, the red line indicates the dose distribution as calculated by MPEXS‐DNA. The dose on the vertical axis indicates the dose value normalized by the total number of incident particles. The dotted green line and dashed blue line indicate the radial dose distributions derived through the use of the analytical calculation models proposed by Chunxiang et al.^21^ and Waligórski et al.^22^ The black dots indicate the measured dose distributions after irradiating a cell‐equivalent gas with protons with 3 MeV of kinetic energy.^19^ The results of MPEXS‐DNA simulations indicate a tendency toward consistency with the experimental data and the results obtained through calculation of the Chunxiang and Waligórski models. Figure 4(b) shows the data obtained with the use of alpha particles with 3 MeV of kinetic energy. Comparison with radial dose distributions obtained through numerical calculations using the Chunxiang and Waligórski models indicates that, in the region of *r *>* *30 nm from the central axis, the dose distributions show major discrepancies with those with MPEXS‐DNA. However, the MPEXS‐DNA dose distribution is close to the behavior observed in the experimental data.^19^ Even in cases of oxygen ions of 2.57 MeV/u, such as those shown in Fig. 6(c), the calculation results for MPEXS‐DNA tend to be consistent with the experimental data^20^ and the numerical calculations using the Chunxiang and Waligórski models. Based on the above results, one can state that the results are consistent with the existing experimental data and the results obtained through numerical calculations, thus confirming the validity of simulations of electromagnetic interactions using the physics stage of MPEXS‐DNA.

### 3.B. Validation of the physicochemical and chemical stages of MPEXS‐DNA

#### 3.B.1. Diffusion and chemical reactions

**Figure 7:**
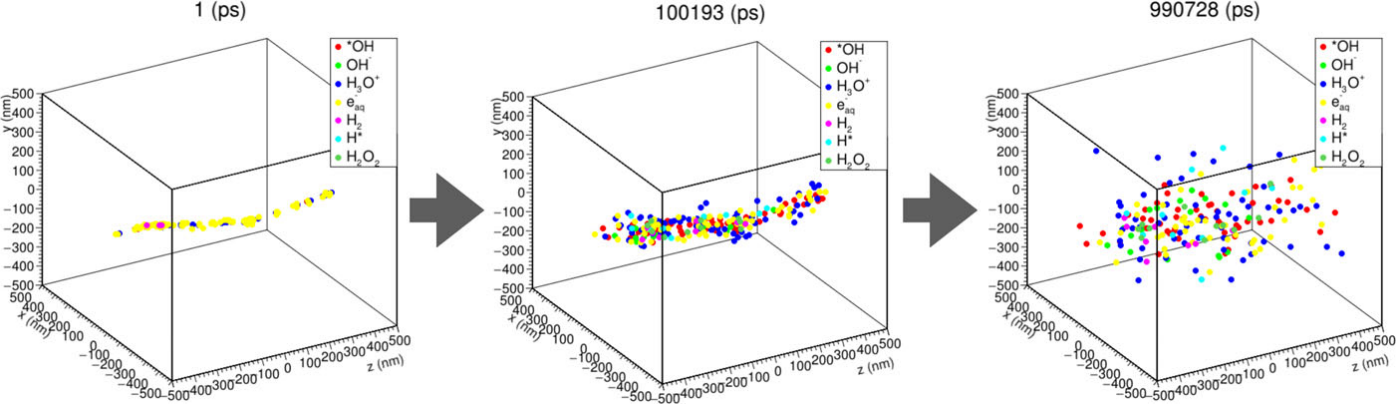
Diffusion and reactions of molecular species induced by 10 keV electrons simulated by MPEXS‐DNA.

Figure 7 shows the diffusion and reactions of molecular species during the period 1 ps to 1 μs after irradiating the water target (size: 1 × 1 × 1 μm^3^) with a 10 keV electron. At 1 ps after irradiation, the molecular species produced are aligned along the track of the electron. Subsequently, as time passes, they repeatedly transform into other molecular species by chemical reactions, and their distribution spreads out.

#### 3.B.2. Comparison of G‐value time profiles and LET dependency with experiment data

##### Comparison of G‐value time profile after irradiation of 750 keV electrons

**Figure 8:**
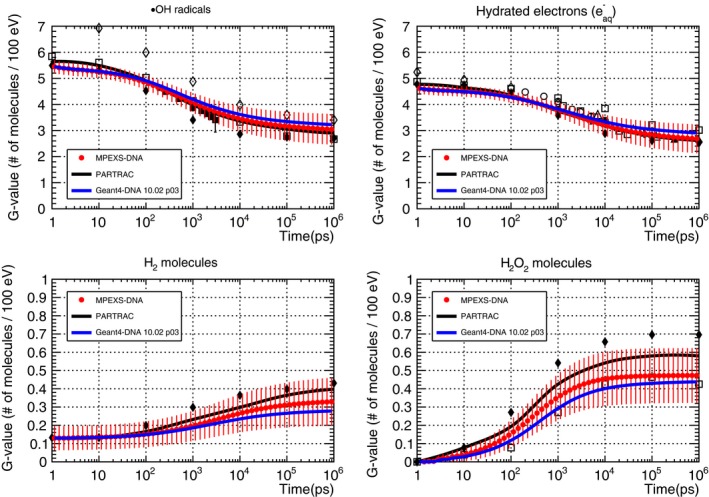
Comparison of G‐value time profiles of ·OH radicals (top left), hydrated electrons (top right), H_2_ molecules (bottom left), and H_2_O_2_ molecules (bottom right) induced by electrons with kinetic energy 750 keV in liquid water. MPEXS‐DNA is represented by filled red circles with vertical bars representing the standard deviation. Geant4‐DNA 10.02 p03 and PARTRAC^23^ are soild blue and black lines, respectively. The other points are experimental and theoretical calculation results (Experimental data: open circles,^28^ filled squares,^29^ open triangles,^30^ filled triangles,^31^ calculations : filled diamonds,^32^ open diamonds,^33^ open squares^34^).

Figure 8 shows our simulation results of the G‐value time profiles for ·OH radicals, hydrated electrons, hydrogen molecules (H_2_), and hydrogen peroxide molecules (H_2_O_2_) between 1 ps and 1 μs after irradiation with 750 keV electrons as well as Geant4‐DNA simulations and experimental data. The red circles show MPEXS‐DNA results. The vertical bars indicate the standard deviations of G‐values. The solid black and blue lines show the results of simulations performed using PARTRAC^23^ and Geant4‐DNA. The other points indicate experimental data and results obtained using numerical calculations (see the caption of Fig. 8 for all references). Hydrated electrons, ·OH radicals, and H_3_O^+^ ions are the major molecular species produced via physical interactions (ionization, excitation, and dissociative electron attachment) during the physicochemical phase. As time passes, the G‐values of these molecular species decrease as they undergo repeated transformations into other molecular species, as a result of diffusion and chemical reactions. H_2_ and H_2_O_2_ molecules are produced by chemical reactions and their G‐values tend to increase with time. For G‐value time profiles of ·OH radicals and hydrated electrons, the simulation results by MPEXS‐DNA are well consistent with the results obtained using PARTRAC. They are also agreements with the experimental data and the results of other numerical calculations. G‐values of H_2_ and H_2_O_2_ molecules by MPEXS‐DNA simulation are lower than PARTRAC and other methods. This is due to the difference in the formation channels for H_2_ and H_2_O_2_ molecules considered in chemical reactions between MPEXS‐DNA and PARTRAC.

Table 15 shows G‐values for all molecular species at 1 ps after irradiation with 750 keV electrons calculated by using each simulation code. MPEXS‐DNA results are in general agreement with the results of other simulation codes. The fact that there are variations in the G‐value data depending on the simulations is possibly due to differences in the physical interactions considered in the physics stage. For example, the G‐values for ·OH radicals and hydrated electrons obtained by PARTRAC are 4–6% higher than those obtained by MPEXS‐DNA. PARTRAC considers the following three physical reactions of electrons: elastic scattering, ionization, and excitation.^23^ In contrast, MPEXS‐DNA considers vibrational excitation and dissociative electron attachment in addition to the above three (see Table 1). As a result, this leads to the discrepancies of the G‐values between two codes.

**Table 15:** Comparison of G‐values at 1 ps after irradiation of 750 keV electrons calculated by MPEXS‐DNA, Geant4‐DNA, PARTRAC23, and other simulation results.^29^, ^30^, ^31^, ^32^, ^33^, ^34^, ^35^, ^36^, ^37^, ^38^

	$e_{\mathrm{aq}}^{-}$	·OH	H·	H_3_O^+^	H_2_
MPEXS‐DNA (this work)	4.61 ± 0.20	5.45 ± 0.25	0.58 ± 0.16	4.63 ± 0.20	0.13 ± 0.07
Geant4‐DNA version 10.02 p03	4.62	5.45	0.58	4.63	0.13
PARTRAC 23	4.83	5.78	0.63	4.83	0.16
Reference 34	4.88	5.89	0.96	–	–
Reference 35	5.30	6.05	0.72	5.38	0.13
Reference 36	4.78	5.70	0.62	4.78	0.15
Reference 37	4.7	6.0	0.8	4.7	0.25
Reference 38	4.78	5.40	0.62	4.78	–
Reference 39	6.3	8.4	2.1	6.3	0.4
Reference 40	–	6.82	0.84	4.8	0.62
Reference 41	4.78	5.50	0.42	4.78	0.15
Reference 42	4.93	5.37	0.45	4.93	0.16
Reference 43	–	5.6 ± 0.3	–	–	–

##### Comparison of G‐value time profile for proton cases

**Figure 9:**
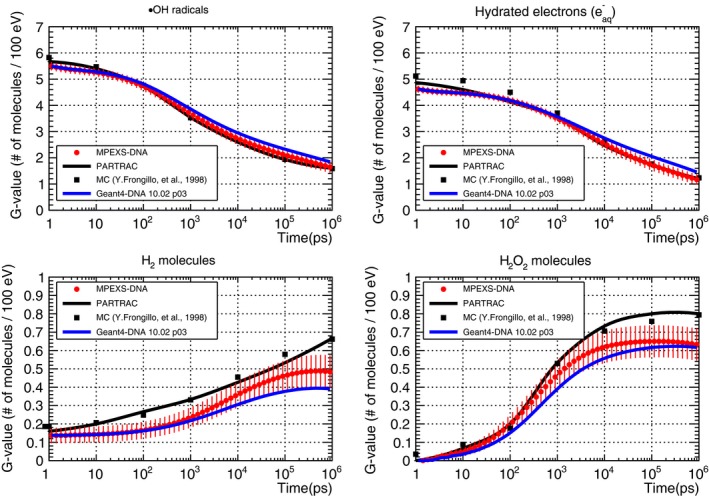
G‐value time profile for ·OH radicals (top left), hydrated electrons (top right), H_2_ (bottom left), and H_2_O_2_ molecules (bottom right) induced by 5 MeV protons. MPEXS‐DNA is represented by filled red circles with vertical bars representing the standard deviation. Solid blue and black lines are Geant4‐DNA 10.02 p03 and PARTRAC^23^ results, respectively. Filled squares are Monte Carlo simulation results by Frongillo et al.^44^

Figure 9 shows the time profiles of the G‐values of ·OH radicals, hydrated electrons, H_2_, and H_2_O_2_ molecules produced when water is irradiated with 5 MeV protons. The results of MPEXS‐DNA calculations are indicated by the red circles with the standard deviations (vertical bars). The solid black and blue lines indicate the results of PARTRAC^23^ and Geant4‐DNA, respectively. The black oblongs indicate the results obtained via the Monte Carlo simulation using the Frongillo et al. method.^44^ The G‐value time profile data for ·OH radicals and hydrated electrons obtained by MPEXS‐DNA agree with PARTRAC and Frongillo et al. simulation results. As mentioned in Section “Comparison of G‐value time profile after irradiation of 750 keV electrons”, differences between MPEXS‐DNA, and PARTRAC and the Frongillo et al. methods in terms of results for H_2_ and H_2_O_2_ molecules are due to the differences in formation channels via chemical reactions for these elements used in each method.

**Figure 10:**
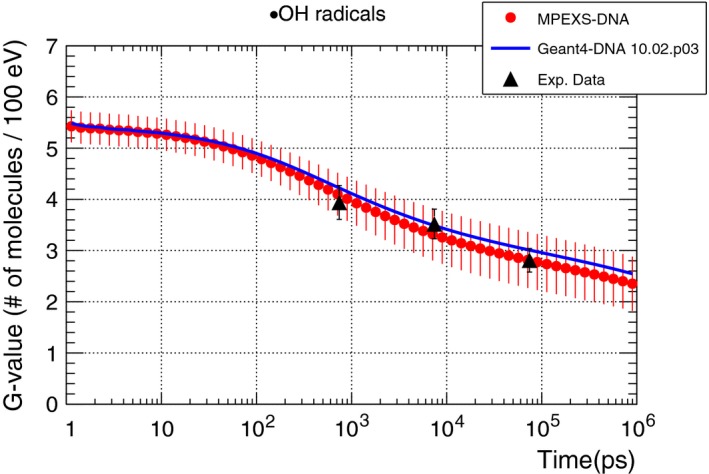
G‐value time profile of ·OH radicals induced by 20 MeV proton in liquid water. Filled red circles are MPEXS‐DNA simulation results with vertical bars representing the standard deviation. Soild blue line is Geant4‐DNA 10.02 p03. Filled triangle is measured data.^45^

Pachnerova et al. irradiated plasmid DNA (pBR322) with proton beams and measured the G‐values of ·OH radicals.^45^ Figure 10 illustrates the comparison of time profiles for ·OH radicals produced by irradiating water with 20 MeV protons, as calculated by MPEXS‐DNA and Geant4‐DNA, and according to the experimental data. In this figure, the red dots indicate the MPEXS‐DNA simulation and the solid blue line indicates Geant4‐DNA simulation. The black triangles indicate the experimental data. The overall data suggest that MPEXS‐DNA can reproduce the experimental results.

##### LET dependency of G‐value

**Figure 11:**
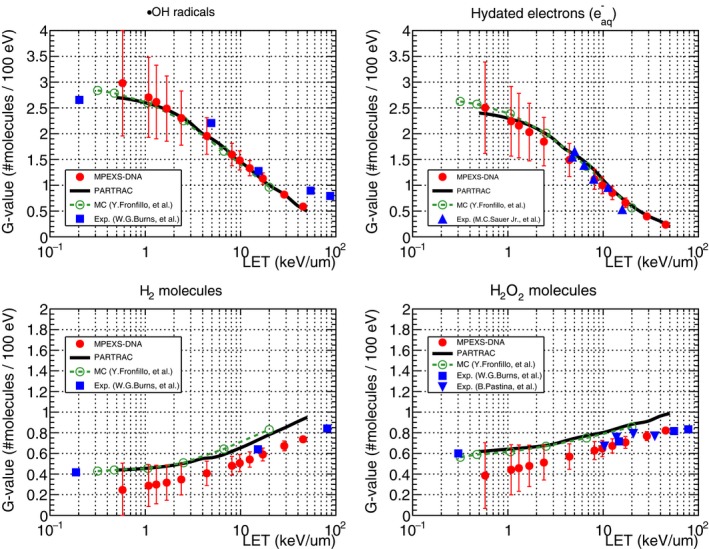
G‐value dependency on LET at 1 μs after irradiation for ·OH radicals, hydrated electrons, H_2_, and H_2_O_2_ molecules. Comparison between simulations irradiating a water target with 500 keV–100 MeV protons and experiments. Filled circles represent MPEXS‐DNA simulations with vertical bars for the standard deviation of G‐value. Solid lines and dashed lines with open circles show the PARTRAC^23^ and Monte Carlo code developed by Frongillo, et al.,^44^ respectively. The other plots are experiment results (filled square,^46^ filled triangle,^47^ and filled inverted triangle^48^).

The LET dependency of G‐values for ·OH radicals, hydrated electrons, H_2_, and H_2_O_2_ were assessed at 1 μs after irradiation of the target with protons (500 keV to 100 MeV). Figure. 11 shows the results of comparison between Monte Carlo simulations, including MPEXS‐DNA and experimental data. In the figure, the red circles indicate MPEXS‐DNA results. The standard deviations of the G‐values are also shown with the vertical bars. The solid black line shows the results of the simulation using PARTRAC.^23^ The dotted line with the open circles indicates the results calculated using the “TRACIRT” Monte Carlo simulator developed by Frongillo, et al.^44^ The oblongs, triangles, and inverted triangles indicate experimental data.^46^, ^47^, ^48^ There is a tendency for the G‐values of ·OH radicals and hydrated electrons to decrease as LET increases, whereas the G‐values for H_2_ and H_2_O_2_ molecules tend to increase as LET increases. This tendency can be interpreted as follows: as LET increases, ·OH radicals and hydrated electrons are produced in proximity to each other during the physics stage (ionization and excitation), and the frequency of chemical reactions increases. Therefore, the G‐values of ·OH radicals and hydrated electrons decrease. Since H_2_ and H_2_O_2_ molecules are the products of chemical reactions from ·OH radicals and hydrated electrons, as LET increases, their G‐values also increase.

The standard deviation of the G‐value for MPEXS‐DNA cases increases as LET decreases. The reason is as follows. In the low LET region, the molecular species are sparsely distributed, and the diffusion distance becomes longer. In addition, the number of molecular species going out of the geometry without chemical reactions increases; thus, the number of molecular species tends to fluctuate.

The results of LET dependency calculations for the G‐values of ·OH radicals and hydrated electrons using MPEXS‐DNA are highly consistent with the results of Monte Carlo simulations using the PARTRAC and TRACIRT codes. In addition, the results obtained from MPEXS‐DNA calculations are generally consistent with the experimental data.^46^, ^47^ However, for H_2_ and H_2_O_2_ molecules, a 20–50% difference between MPEXS‐DNA and both PARTRAC and TRACIRT is found. This is caused by differences between the various simulation codes in terms of molecular species and chemical reactions considered. In particular, TRACIRT handles twice as many types of molecular species as MPEXS‐DNA and five more chemical reactions. For example, regarding the formation channels of H_2_O_2_, MPEXS‐DNA considers only a single channel ($\cdot \text{OH}+\cdot \text{OH}\to H_{2}O_{2}$), whereas TRACIRT considers a total of six. Since both PARTRAC and TRACIRT produce results similar to the experimental data,^46^, ^48^ MPEXS‐DNA requires further improvement on this particular point.

### 3.C Benchmark results of MPEXS‐DNA

MPEXS‐DNA is a track structure and radiolysis simulator that utilizes a GPU. Through the use of ultraparallel processing, it can work at higher speeds than conventional simulations that utilize a CPU and it can calculate energy loss distributions and molecular species distributions in liquid water. MPEXS‐DNA is based on Geant4‐DNA, and as indicated in Section 2.C.4, its results are highly consistent with those of Geant4‐DNA. Geant4‐DNA has an issue with long duration simulation time. Due to the fact that processing an enormous number of secondary particles and molecular species using the Monte Carlo method in order to calculate both local energy loss and molecular species distributions with higher accuracy, Geant4‐DNA, as mentioned in the chapter 1, requires long time for simulations even on a CPU cluster. Comparison of the computing performance achieved by MPEXS‐DNA to Geant4‐DNA is thus of great significance.

Table 16 shows the calculation environment utilized in our benchmark. TITAN V, a GPU device manufactured by NVIDIA, was utilized in the MPEXS‐DNA benchmark. To maximize the computing performance of MPEXS‐DNA, L1 cache was enabled, and the fast‐math option, which ensures specialized calculations such as trigonometric and logarithm functions at high speed, was used. The Geant4‐DNA performance was measured using a single Intel^®^ Xeon^®^ CPU (6‐core model). The time required for initialization that was performed prior to the simulation was negligible compared to the simulation time in both Geant4‐DNA and MPEXS‐DNA. In these benchmark tests, the initialization time was excluded from both the datasets, so that only the processing time from the physics stage to the chemical stage was assessed. The throughput of both MPEXS‐DNA and Geant4‐DNA simulations was evaluated with the number of events processed per minute. The definition of “one event” corresponds to the entire process including physics and chemical stages of irradiation by an initial particle. Tables 17, 18, 19 present the benchmark results for the cases of 750 keV electrons as well as both 5 and 20 MeV protons, respectively.

**Table 16:** Computing system utilized for benchmark test of MPEXS‐DNA

	CPU/GPU/Workstation	OS/GCC/CUDA
MPEXS‐DNA	Intel^®^ Xeon^®^ Gold 6132 2.60 GHz/NVIDIA^®^ TITAN V 1,455 MHz, 5,120 cores, 12 GB HBM2/HP Z8	CentOS Linux 7.4.1708/GCC 4.8.5/CUDA 9.2.88
Geant4‐DNA	Intel^®^ Xeon^®^E5‐2643 v2 3.50 GHz/HP Z820	CentOS Linux 7.4.1708 GCC 4.8.5

**Table 17:** Benchmark results for 750 keV electron irradiation between Geant4‐DNA 10.02 p03 (CPU) and MPEXS‐DNA (GPU)

	Geant4‐DNA	MPEXS‐DNA
Initial particle	Electrons with kinetic energy of 750 keV	
Target size	20 × 20 × 20 μm^3^	
Number of voxels in the target	A single voxel	
Throughput (the physics stage)	2667.4	337 868.8
Speedup factor	–	126.6
Throughput (the chemical stage)	1.155	1724.1
Speedup factor	–	1492
Throughput (the physics and the chemical stages)	1.154	1715.3
Speedup factor	–	1486

**Table 18:** Benchmark results for 5 MeV proton irradiation between Geant4‐DNA 10.02 p03 (CPU) and MPEXS‐DNA (GPU)

	Geant4‐DNA	MPEXS‐DNA
Initial particle	Protons with kinetic energy of 5 MeV	
Target size	1 × 1 × 1 μm^3^	
Number of voxels in the target	A single voxel	
Throughput (the physics stage)	1155.8	615 902.2
Speedup factor	–	532.9
Throughput (the chemical stage)	0.265	536.04
Speedup factor	–	2023
Throughput (the physics and the chemical stages)	0.264	535.58
Speedup factor	–	2029

**Table 19:** Benchmark results irradiating a cubic water target with 20 MeV protons comparing Geant4‐DNA 10.02 p03 (CPU) and MPEXS‐DNA (GPU)

	Geant4‐DNA	MPEXS‐DNA
Initial particle	Protons with kinetic energy of 20 MeV	
Target size	1 × 1 × 1 μm^3^	
Number of voxels in the target	A single voxel	
Throughput (the physics stage)	3986.0	1 111 059.9
Speedup factor	–	278.7
Throughput (the chemical stage)	2.624	7728.7
Speedup factor	–	2945
Throughput (the physics and the chemical stages)	2.622	7675.2
Speedup factor	–	2927

In all three tables, the initial particle, the target size, throughput, and speedup factor against Geant4‐DNA are indicated for each benchmark test. In comparison with Geant4‐DNA simulation with single core of CPU, MPEXS‐DNA has achieved speeds that are 2900 times faster at maximum. The cost of the GPU in this work is about the same as CPU processors used in workstations. A GPU is a very cost‐effective tool for accelerating our simulation.

## 4 Conclusion

MPEXS‐DNA is a new track structure and radiolysis simulation code that is based on the Geant4‐DNA package available in Geant4 10.02 p03. It simulates the electromagnetic interactions of charged particles in liquid water, calculates the track structure of particles in a microregion at nanometer level, and allows calculation of the localized energy loss distribution. In addition, it simulates diffusion and chemical reactions for molecular species produced by the radiolysis of water and allows the determination of the distribution of molecular species within a target region. In this paper, initial particles such as electrons and protons were used to irradiate a water target, and the energy loss distributions and G‐value time profiles for molecular species were calculated. We have confirmed that the simulation results obtained by MPEXS‐DNA are consistent with the existing experimental data and simulations performed using state‐of‐the‐art codes such as PARTRAC, which indicates the validity of simulations performed by the MPEXS‐DNA. MPEXS‐DNA is a Monte Carlo simulator that utilizes a GPU. Compared with simulations performed by Geant4‐DNA with a single CPU core, MPEXS‐DNA can perform simulations 2900 times faster at maximum with the same accuracy as Geant4‐DNA. MPEXS‐DNA dramatically improved the computational speed of Geant4‐DNA. In the future, the biological phase will be implemented to quantitatively estimate the cell survival rate as well as DNA damage including strand breaks and base damage.

## Conflicts of interest

The authors have no relevant conflicts of interest to disclose.
